# A Comparative Study of OCR Architectures for Korean License Plate Recognition: CNN–RNN-Based Models and MobileNetV3–Transformer-Based Models

**DOI:** 10.3390/s26041208

**Published:** 2026-02-12

**Authors:** Seungju Lee, Gooman Park

**Affiliations:** Department of Smart ICT Convergence Engineering, Seoul National University of Science and Technology, Seoul 01811, Republic of Korea; sjleee05@gmail.com

**Keywords:** license plate recognition, OCR, transformer, attention-LSTM, embedded vision

## Abstract

This paper presents a systematic comparative study of optical character recognition (OCR) architectures for Korean license plate recognition under identical detection conditions. Although recent automatic license plate recognition (ALPR) systems increasingly adopt Transformer-based decoders, it remains unclear whether performance differences arise primarily from sequence modeling strategies or from backbone feature representations. To address this issue, we employ a unified YOLOv12-based license plate detector and evaluate multiple OCR configurations, including a CNN with an Attention-LSTM decoder and a MobileNetV3 with a Transformer decoder. To ensure a fair comparison, a controlled ablation study is conducted in which the CNN backbone is fixed to ResNet-18 while varying only the sequence decoder. Experiments are performed on both static image datasets and tracking-based sequential datasets, assessing recognition accuracy, error characteristics, and processing speed across GPU and embedded platforms. The results demonstrate that the effectiveness of sequence decoders is highly dataset-dependent and strongly influenced by feature quality and region-of-interest (ROI) stability. Quantitative analysis further shows that tracking-induced error accumulation dominates OCR performance in sequential recognition scenarios. Moreover, Korean license plate–specific error patterns reveal failure modes not captured by generic OCR benchmarks. Finally, experiments on embedded platforms indicate that Transformer-based OCR models introduce significant computational and memory overhead, limiting their suitability for real-time deployment. These findings suggest that robust license plate recognition requires joint consideration of detection, tracking, and recognition rather than isolated optimization of OCR architectures.

## 1. Introduction

With the advancement of intelligent transportation systems (ITS), automatic license plate recognition (ALPR) has become a core component in applications such as traffic law enforcement, parking management, and public safety. Recent progress in deep learning–based object detection and optical character recognition (OCR) has enabled ALPR systems to achieve high accuracy even under complex outdoor conditions.

**Figure 1:**
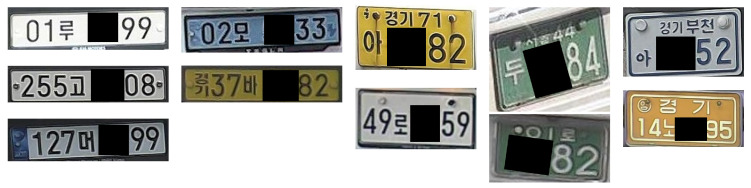
Diversity of Korean license plates across different formats, layouts, and character compositions, illustrating variations in regional prefixes, Korean characters, alphanumeric structures, and visual appearance under real-world conditions. The Korean characters shown in the figure (e.g., regional names and vehicle classification symbols) are part of the official license plate system.

Despite this progress, Korean license plate recognition remains particularly challenging. Korean license plates contain mixed Korean characters and numerals, regional prefixes, and diverse color schemes and layouts, often combined with reflective materials that cause optical distortions, as illustrated in Figure 1. In real traffic environments, additional factors such as illumination variation, long-distance capture, motion blur, and low-resolution imagery further degrade recognition performance.

Most prior ALPR studies have focused on improving detection accuracy or optimizing standalone OCR models. In recent years, two-stage pipelines combining YOLO-family detectors with recurrent neural network (RNN)- or Transformer-based OCR models [1,2] have become common. However, existing studies rarely provide controlled comparisons of different OCR architectures under identical detection results. As a result, it remains unclear whether observed performance differences originate from sequence modeling strategies or from differences in backbone feature representations, particularly in the context of Korean license plates.

To address this gap, this study investigates a two-stage license plate recognition pipeline by systematically comparing two OCR architectures with distinct design philosophies. Specifically, license plate regions detected by a YOLOv12-based detector [3] are fed into (1) a conventional OCR model based on convolutional neural network (CNN) feature extraction and Attention-LSTM sequence modeling [4,5] and (2) a lightweight OCR model combining MobileNetV3 [6] with a Transformer decoder. To ensure a fair comparison, the detector outputs are fixed and shared across all OCR models.

Through extensive experiments on multiple public datasets and real-world CCTV data, this work evaluates recognition accuracy, error characteristics, and processing speed across both GPU and embedded platforms. By analyzing the conditions under which different OCR architectures succeed or fail, this study aims to clarify the practical limitations of Transformer-based OCR models and to provide design insights for robust, real-time ALPR systems operating in real-world traffic environments.

The main contributions of this study are summarized as follows:

- Empirical validation of tracking-induced OCR failure modes: Unlike most prior OCR studies that focus on single-frame recognition accuracy, this work provides quantitative evidence that tracking-induced ROI instability (jitter, drift, and frozen regions) is the dominant factor governing recognition failure in sequential license plate datasets.
- Korean license plate–specific error analysis beyond generic OCR metrics: We introduce and quantitatively analyze error categories unique to Korean license plates, including prefix misrecognition and structural collapse, which are not addressed in existing Latin-based ALPR benchmarks.
- Controlled ablation-based comparison of OCR decoders: By fixing the CNN backbone and varying only the sequence decoder, we demonstrate that decoder choice alone does not explain performance differences, particularly under tracking-based conditions.
- Quantitative assessment of Transformer-based OCR limitations in embedded environments: Through model complexity analysis and multi-platform experiments, we show that Transformer-based OCR models incur higher FLOPs and memory overhead, leading to severe FPS degradation on embedded devices despite their architectural parallelism.
- Practical design insights for real-world ALPR systems: The combined findings indicate that robust license plate recognition requires integrated optimization across detection, tracking, and recognition stages rather than isolated improvements to OCR architectures.

## 2. Related Works

### 2.1. Background of License Plate Detection

License plate detection constitutes the first stage of an automatic license plate recognition (ALPR) system and directly influences overall recognition performance. Early approaches relied on hand-crafted features such as Haar-like features [7], Histogram of Oriented Gradients (HOG) [8], and Support Vector Machines (SVM) [9]. With the advent of deep learning, convolutional neural network (CNN)-based object detectors [10,11] have become the dominant paradigm for license plate detection.

Among these, the YOLO (You Only Look Once) [12] family has been widely adopted due to its single-stage architecture, which offers an effective trade-off between detection accuracy and inference speed. Successive versions, including YOLOv3 [13], YOLOv5 [14], and YOLOv8 [15], have progressively improved detection robustness and efficiency. More recently, YOLOv12 has been introduced with architectural refinements and optimized training strategies, enabling flexible deployment across lightweight and medium-scale configurations.

Most prior studies primarily evaluate detection accuracy under idealized settings. In contrast, recent works have increasingly emphasized deployment-aware evaluation, considering inference frameworks and hardware constraints. Following this trend, YOLOv12-based detectors are commonly assessed at fixed input resolutions and optimized inference backends to better reflect real-world ALPR system requirements.

### 2.2. Lightweight Vision Backbones

ALPR systems are frequently deployed in traffic infrastructure and edge-device environments, where computational efficiency and memory footprint are critical constraints. As a result, lightweight convolutional backbones [16,17,18] have been extensively studied for practical license plate recognition pipelines. The MobileNet family represents one of the most widely adopted classes of efficient CNN architectures in this context.

MobileNetV1 [19] introduced depthwise separable convolutions to reduce computational cost, while MobileNetV2 [20] improved representational capacity through inverted residual blocks and linear bottlenecks. MobileNetV3 further optimized the accuracy–efficiency trade-off by incorporating Neural Architecture Search (NAS) [21] and Squeeze-and-Excitation (SE) [22] modules. Owing to these characteristics, MobileNet-based backbones have been widely used in real-time OCR and ALPR systems.

While prior studies have explored various combinations of lightweight backbones and sequence decoders, most evaluations focus on architectural performance in isolation. Comparative analyses that control detection and tracking outputs while examining OCR behavior under sequential, real-world conditions remain limited, particularly for non-Latin license plate systems such as those used in Korea.

## 3. Proposed Method

This study adopts a two-stage pipeline for Korean license plate recognition, in which license plate detection and optical character recognition (OCR) are explicitly separated. The system first localizes license plate regions from input images and subsequently applies different OCR models to normalized license plate crops for comparative analysis.

### 3.1. Overall System Architecture

**Figure 2:**
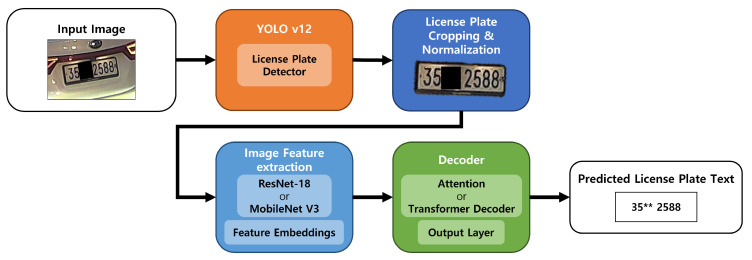
Overall architecture of the proposed license plate recognition system, consisting of detection, tracking, and OCR modules. End-to-end latency is determined by the cumulative processing time of these stages, while OCR performance is analyzed independently in this study. The symbol “**” indicates regions intentionally masked to protect personal information.

Figure 2 illustrates the overall architecture of the proposed license plate recognition system. Given an input image, a YOLOv12-based detector first localizes license plate regions. The detected regions are then cropped and normalized to a fixed input format suitable for OCR. The normalized license plate images are finally processed by the recognition model to generate the predicted character sequence.

By decoupling detection and recognition, the proposed architecture enables independent optimization of each stage and facilitates controlled evaluation of OCR model behavior under identical detection outputs.

#### Tracking Module and Scope Definition

In sequential recognition scenarios, a lightweight tracking module is employed to propagate detected license plate regions across consecutive frames. In this study, tracking is intentionally treated as a region-of-interest (ROI) propagation mechanism rather than as a primary research target. Specifically, a simple IoU-based association strategy is adopted to link license plate detections across frames, following common SORT-like tracking paradigms. Representative and standard parameter settings are used to reflect typical deployment configurations, without task-specific fine-tuning of the tracking component. It should be emphasized that the tracking module is not independently optimized or quantitatively evaluated in terms of tracking accuracy (e.g., precision or recall), as such analysis lies outside the scope of this work. Instead, the tracking stage is used to model realistic ROI propagation behavior in practical ALPR systems, enabling system-level analysis of how tracking-induced ROI instability affects downstream OCR performance.

### 3.2. License Plate Detection

In the detection stage, YOLOv12-based models are employed to localize license plate regions. Three model scales—YOLOv12-n, YOLOv12-s, and YOLOv12-m—are trained to analyze the trade-off between detection accuracy and inference efficiency across different model sizes. All detectors are trained using a fixed input resolution of 640×640.

To reflect practical deployment conditions, the trained models are exported to TensorRT and TensorFlow Lite (TFLite) formats and evaluated on multiple hardware platforms. This setup enables assessment of both detection accuracy and inference speed (FPS) under realistic system constraints relevant to real-time license plate recognition.

### 3.3. License Plate Recognition Models

#### 3.3.1. CNN + Attention-LSTM-Based OCR (v4)

**Figure 3:**
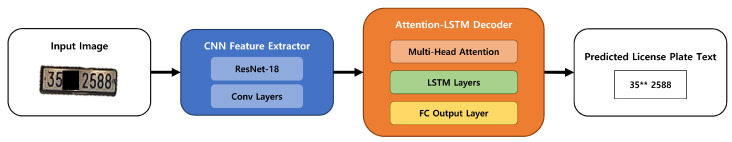
CNN + Attention-LSTM-based OCR model (v4), which employs a convolutional feature extractor followed by an attention-guided LSTM decoder for sequential license plate character recognition. The symbol “**” indicates regions intentionally masked to protect personal information.

The first OCR model, shown in Figure 3, follows a conventional sequence recognition architecture that combines a convolutional neural network (CNN) feature extractor with an Attention-based LSTM decoder. The model transforms spatial feature maps into a sequence representation and predicts license plate characters sequentially by modeling inter-character dependencies.

##### CNN Feature Extractor

A ResNet-18 backbone [23] pretrained on ImageNet is used as the feature extractor. The final classification layer is removed, and the resulting convolutional feature maps are used as input to the sequence decoder. Batch normalization is applied to the output feature maps to improve training stability.

The CNN output has an initial shape of (B,C,H,W) and is rearranged to (B,H,W,C) to preserve the spatial ordering of characters along the horizontal axis of the license plate. This representation facilitates subsequent conversion into a character sequence.

##### Attention-Based Decoder

The decoder consists of a self-attention module followed by an LSTM network. The two-dimensional feature maps are flattened into a sequence, and self-attention is applied to capture global contextual relationships among character features. The LSTM then processes the attention-enhanced sequence to predict characters sequentially. At each time step, the decoder output is passed through a fully connected layer to produce character class probabilities.

##### Model Characteristics

The CNN + Attention-LSTM architecture provides a structured approach to sequence modeling by combining spatial feature extraction with recurrent decoding. While the LSTM-based decoder processes characters sequentially, which limits parallelism during inference, the overall architecture remains computationally efficient for short character sequences such as license plates.

#### 3.3.2. MobileNetV3 + Transformer Decoder-Based OCR (v7)

**Figure 4:**
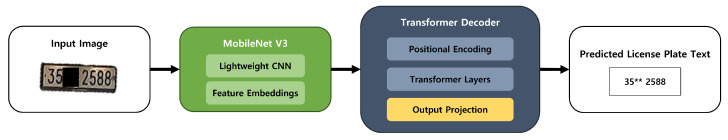
MobileNetV3 + Transformer decoder-based OCR model (v7), where a lightweight CNN backbone extracts visual features and a Transformer decoder models global character dependencies for robust license plate recognition. The symbol “**” indicates regions intentionally masked to protect personal information.

The second OCR model, illustrated in Figure 4, combines a lightweight CNN-based feature extractor with a Transformer decoder. The model is designed to leverage self-attention for global dependency modeling while maintaining computational efficiency through a lightweight backbone.

##### CNN Feature Extractor: MobileNetV3

MobileNetV3-Large is used as the feature extractor due to its favorable accuracy–efficiency trade-off. The network employs inverted bottleneck structures and Squeeze-and-Excitation (SE) modules to reduce computational cost while preserving representational capacity. The extracted feature maps with shape (B,C,H,W) are transformed into a width-wise sequence representation of shape (W,B,D), which aligns with the left-to-right character ordering of license plate text.

##### Transformer Decoder

The Transformer decoder consists of token embedding layers, positional encoding, and multiple stacked decoder layers. Masked self-attention is applied to model contextual dependencies within the output sequence, while encoder–decoder attention integrates visual features extracted by the CNN. The decoder outputs are projected through a linear layer to produce character class probabilities.

##### Positional Encoding

To encode the sequential order of characters, a fixed sinusoidal positional encoding scheme is adopted. Given the short and bounded length of license plate strings, fixed positional encoding provides sufficient positional discrimination without introducing additional learnable parameters. Preliminary experiments with learnable positional embeddings did not yield consistent accuracy improvements and increased model complexity; therefore, fixed positional encoding is used for simplicity and deployment efficiency.

##### Model Characteristics

The Transformer-based decoder enables global context modeling through self-attention. However, under batch-size-one, autoregressive decoding—common in real-time ALPR deployments—the decoding process remains sequential across time steps. In addition, self-attention operations and decoder-side projections introduce non-negligible computational and memory overhead, particularly on embedded platforms with limited memory bandwidth. Consequently, the practical inference speed of the v7 model depends strongly on hardware characteristics and deployment settings rather than architectural parallelism alone.

### 3.4. Model Comparison and Experimental Objective

The structural differences between the two OCR models are summarized in Table 1. The CNN + Attention-LSTM model adopts a recurrent sequence modeling strategy with a conventional CNN backbone, while the MobileNetV3 + Transformer model combines a lightweight feature extractor with self-attention-based decoding.

In this study, identical license plate detection results are provided as input to both recognition models to isolate OCR behavior from detection variability. Performance is evaluated in terms of recognition accuracy (CA, CER, and edit distance) and inference speed (FPS). This controlled setting enables quantitative analysis of how different sequence modeling strategies affect recognition performance under static and tracking-based conditions in Korean license plate recognition.

### 3.5. Model Complexity Analysis

The model size and computational complexity of the evaluated OCR architectures are summarized in Table 2. Although the Transformer-based OCR model has fewer parameters than the CNN + Attention-LSTM model, its overall computational cost is higher due to the quadratic complexity of self-attention operations and additional decoder-side projections. Furthermore, the larger activation memory required by the Transformer decoder introduces non-negligible latency on memory-bandwidth-limited embedded platforms. These structural characteristics explain the discrepancy between parameter count and practical inference speed, particularly under batch-size-one, autoregressive decoding conditions commonly encountered in real-time ALPR systems.

### 3.6. Controlled Ablation for Fair Decoder Comparison

In the primary comparison, the two OCR models differ in both CNN backbone and sequence decoder, which complicates attribution of performance differences to the decoder design alone. To disentangle these factors, we conduct a controlled ablation study in which the CNN backbone is fixed and only the sequence decoder is varied. It should be clarified that this ablation does not constitute a strictly isolated decoder-only comparison in the theoretical sense, as the sequence decoder inevitably operates on features shaped by the preceding backbone. However, by enforcing a shared representational constraint through a fixed ResNet-18 feature extractor, this setup provides a controlled and practically fair proxy for comparing sequence decoding strategies. Importantly, the feature-to-sequence transformation—specifically, the flattening and width-wise representation of convolutional feature maps—is applied identically across all ablation configurations. As a result, both decoders receive equivalent spatial and sequential inputs, ensuring that any observed performance differences arise from the decoder design itself rather than from differences in feature representation or input formatting.

Specifically, a ResNet-18 feature extractor pretrained on ImageNet is paired with an Attention-LSTM decoder, following the same feature-to-sequence transformation used in the main experiments. All training and inference conditions—including input resolution, character set, maximum sequence length, and optimization settings—are kept identical.

This ablation isolates the impact of sequence modeling while eliminating backbone-induced variations, enabling a fair assessment of decoder-level contributions to license plate recognition performance.

## 4. Experiments

### 4.1. Dataset and Experimental Settings

This section describes the datasets, experimental environments (hardware and software), and evaluation metrics used to assess the proposed Korean license plate recognition system. All experiments are conducted using a two-stage pipeline in which license plate detection and recognition are explicitly separated.

Performance is evaluated not only in terms of recognition accuracy but also with respect to processing speed, in order to assess the practical applicability of each configuration in real-world ALPR deployments.

#### 4.1.1. Datasets

To evaluate recognition performance under diverse imaging conditions and license plate structures, three datasets are used in this study: UFPR-ALPR [24], RodoSol-ALPR [25], and the AI-HUB Vehicle and License Plate Recognition Dataset [26]. These datasets differ in acquisition environments, camera configurations, and license plate formats, enabling assessment of both cross-domain generalization and Korean-specific recognition characteristics.

##### UFPR-ALPR Dataset

UFPR-ALPR is a public ALPR dataset collected in real driving scenarios where both vehicles and cameras are in motion, resulting in realistic capture conditions. The dataset contains 4500 images from 150 vehicles, with annotations covering more than 30,000 license plate characters. Images are captured using three cameras—GoPro Hero4 Silver, Huawei P9 Lite, and iPhone 7 Plus—and provided in PNG format at a resolution of 1920×1080. Each camera contributes 1500 images, allowing analysis of domain shifts caused by camera-specific characteristics.

The dataset includes gray-license-plate cars (900 images), red-license-plate cars (300 images), and gray-license-plate motorcycles (300 images). Data are split into training, validation, and test sets with ratios of 40%, 20%, and 40%, respectively. Motion blur, illumination variation, and viewpoint changes make UFPR-ALPR a challenging benchmark, particularly for OCR under degraded conditions.

##### RodoSol-ALPR Dataset

RodoSol-ALPR is a large-scale dataset collected using fixed cameras at toll booths along the ES-060 highway in Espírito Santo, Brazil. It consists of 20,000 images covering multiple vehicle types, including cars, motorcycles, buses, and trucks.

All images have a resolution of 1280×720 and include both Brazilian legacy and Mercosur license plate standards. The dataset is split into training, validation, and test sets at ratios of 40%, 20%, and 40%, respectively, with annotations provided as four corner coordinates of license plates.

In this study, RodoSol-ALPR is used exclusively as a cross-domain generalization benchmark. Given its Latin-based alphanumeric license plate structure, it is not used for Korean-specific error analysis, which is instead conducted primarily on the AI-HUB dataset.

##### AI-HUB Vehicle and License Plate Recognition Dataset

The AI-HUB dataset is a large-scale video-based dataset collected from real-world CCTV environments in South Korea. It comprises over 500 h of video captured at 75 locations and includes approximately 500,000 vehicle images and 100,000 license plate images with JSON-based annotations.

This dataset reflects characteristics unique to Korean license plates, including mixed digit–Korean–digit structures, multiple layout variants, color-based categories, and reflective distortions caused by plate materials and illumination conditions. These properties make the dataset particularly suitable for evaluating Korean ALPR systems.

In this study, the AI-HUB dataset serves as the primary benchmark for analyzing Korean license plate–specific recognition characteristics, such as prefix handling, structural consistency, and region-dependent layout variations. Accordingly, all Korean-specific error analyses reported in this work are based primarily on experiments conducted using the AI-HUB dataset.

#### 4.1.2. Hardware and Software Environment

Experiments are conducted in both server-class GPU environments and embedded device environments to evaluate performance across diverse deployment scenarios. The server setup uses an NVIDIA GeForce RTX 2080 Ti GPU (NVIDIA Corporation, Santa Clara, CA, USA), while embedded platforms include the Jetson Nano (NVIDIA Corporation, Santa Clara, CA, USA) and Raspberry Pi 5 (Raspberry Pi Ltd., Cambridge, UK).

License plate detection models are implemented using Ultralytics YOLOv12 within Docker-based environments. Recognition models are trained and evaluated using PyTorch 2.1.0 (Meta AI, Menlo Park, CA, USA)-based Docker containers.

Software versions and inference backends vary across platforms to reflect realistic deployment conditions in practical ALPR systems.

#### 4.1.3. Evaluation Metrics

Performance is evaluated separately for the detection and recognition stages. In addition to accuracy-related metrics, processing speed measured in frames per second (FPS) is reported to assess computational efficiency.

##### Detection Metrics

Detection performance is evaluated using precision, recall, and F1-score. A detection is considered correct if the Intersection over Union (IoU) between the predicted and ground-truth bounding boxes exceeds a predefined threshold. FPS is reported to characterize detection efficiency under different hardware environments.

##### Recognition Metrics

Recognition performance is evaluated using edit distance (ED), character accuracy (CA), and character error rate (CER). Recognition FPS is also reported to assess the real-time feasibility of each OCR model.

End-to-end latency is discussed at a conceptual level by decomposing the ALPR pipeline into detection, ROI cropping and normalization, tracking, OCR inference, and post-processing stages. In this study, detection and recognition stages are evaluated independently to enable controlled comparison of OCR architectures under identical detection results. Consequently, direct end-to-end latency measurements of a fully integrated runtime pipeline are not reported, and this is treated as a limitation of the experimental scope.

### 4.2. Training and Inference Details for OCR Models

Table 3 summarizes the training and inference settings used for all OCR models evaluated in this study. To ensure fair comparison, identical training protocols and inference configurations were applied across architectures unless explicitly stated otherwise.

All OCR models were trained using identical character vocabularies and maximum sequence length constraints to ensure comparability. Autoregressive decoding was adopted to reflect realistic deployment conditions, where OCR is applied independently to each detected license plate region without temporal integration. During inference, greedy decoding was consistently used to avoid confounding effects introduced by beam search or language-model-based post-processing.

To improve statistical reliability, all OCR experiments were repeated using different random seeds. Unless otherwise stated, reported recognition results correspond to the mean values obtained from three independent training runs. Standard deviation is reported alongside mean values where applicable to indicate performance variability. This protocol reduces the influence of stochastic factors such as weight initialization and data shuffling, enabling a more stable and fair comparison across OCR architectures.

### 4.3. License Plate Detection Results

This subsection evaluates license plate detection performance using nine YOLOv12-based models across different datasets and hardware platforms.

#### 4.3.1. Detection Performance on RTX 2080 Ti

**Figure 5:**
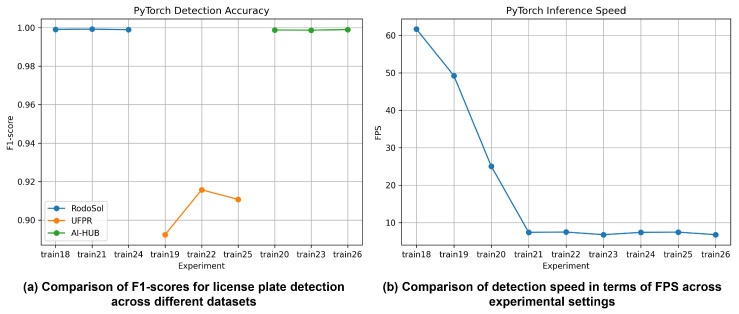
License plate detection performance using a PyTorch-based implementation on an RTX 2080 Ti GPU: (**a**) F1-score comparison across datasets; (**b**) detection speed (FPS) comparison across experimental settings.

Detection performance is first evaluated using the original PyTorch models on an RTX 2080 Ti GPU. As shown in Figure 5, models trained on the RodoSol-ALPR and AI-HUB datasets (train18, train20, train21, train23, train24, and train26) achieve consistently high detection accuracy, with precision, recall, and F1-score values exceeding 0.998 across all configurations. The minimal gap between precision and recall indicates stable detection behavior with few false positives or missed detections.

In contrast, models trained on the UFPR-ALPR dataset (train19, train22, and train25) exhibit lower detection performance, with F1-scores ranging from approximately 0.89 to 0.92. This degradation can be attributed to the challenging characteristics of the UFPR dataset, including large viewpoint variations, illumination changes, and motion blur, which aligns with observations reported in previous studies.

While detection accuracy varies only marginally across model sizes, inference speed differs substantially. YOLOv12-n-based models (train18–train20) achieve real-time performance exceeding 60 FPS, whereas YOLOv12-s and YOLOv12-m models (train21–train26) show significantly lower speeds of approximately 6–7 FPS. Based on this trade-off, YOLOv12-n-based models (train18, train19, and train20) are selected as the license plate detectors in the proposed system, as they offer a favorable balance between detection accuracy and computational efficiency.

#### 4.3.2. TensorRT-Based Optimization

**Figure 6:**
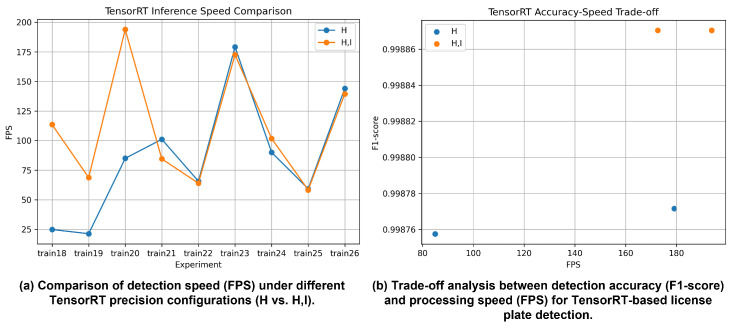
License plate detection performance using a TensorRT-optimized implementation on an RTX 2080 Ti GPU, where (**a**) compares the detection speed (FPS) under different TensorRT precision configurations (H vs. H,I) and (**b**) analyzes the trade-off between detection accuracy (F1-score) and processing speed (FPS).

Figure 6 illustrates the performance changes observed when the detection models are converted to TensorRT engines with different precision configurations. On the RTX 2080 Ti, FP16 TensorRT inference maintains precision, recall, and F1-score values comparable to those of the original PyTorch models, while substantially improving inference speed. For instance, models trained on the AI-HUB dataset (train20, train23, and train26) achieve FPS values of up to approximately 190, satisfying real-time deployment requirements.

In contrast, INT8 quantization leads to notable drops in precision for several models and datasets, including train18, train21, and train24. This degradation indicates that aggressive quantization can adversely affect boundary-sensitive tasks such as license plate detection. These results suggest that while FP16 TensorRT optimization provides a reliable speed–accuracy trade-off, the use of INT8 quantization requires careful calibration and dataset-specific validation rather than indiscriminate application.

#### 4.3.3. TensorFlow Lite Inference Performance

**Figure 7:**
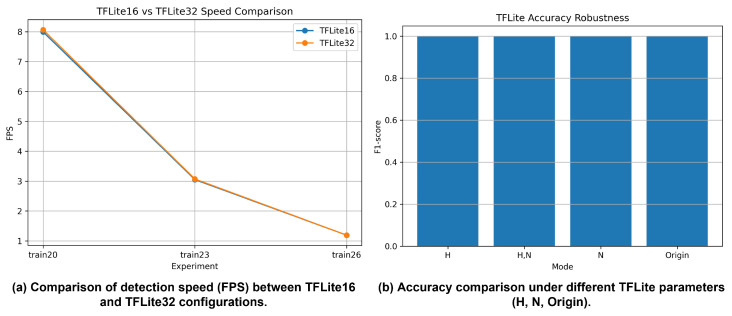
License plate detection performance using TensorFlow Lite on an RTX 2080 Ti GPU: (**a**) detection speed (FPS) comparison between FP16 (TFLite16) and FP32 (TFLite32) configurations and (**b**) detection accuracy under different TensorFlow Lite parameter settings.

TensorFlow Lite-based inference is evaluated using FP16 (TFLite16) and FP32 (TFLite32) configurations with different runtime options, as shown in Figure 7. On the RTX 2080 Ti, TensorFlow Lite models maintain precision, recall, and F1-score values comparable to those of the original PyTorch models, indicating that detection accuracy is largely preserved.

However, inference speed achieved with TensorFlow Lite is consistently lower than that of TensorRT-based inference. In addition, FPS degradation becomes more pronounced as model size increases, reflecting structural limitations in fully exploiting GPU acceleration. These results suggest that while TensorFlow Lite is effective for maintaining detection accuracy, it is less suitable than TensorRT for high-throughput GPU-based deployment.

#### 4.3.4. Detection Performance on Jetson Nano

**Figure 8:**
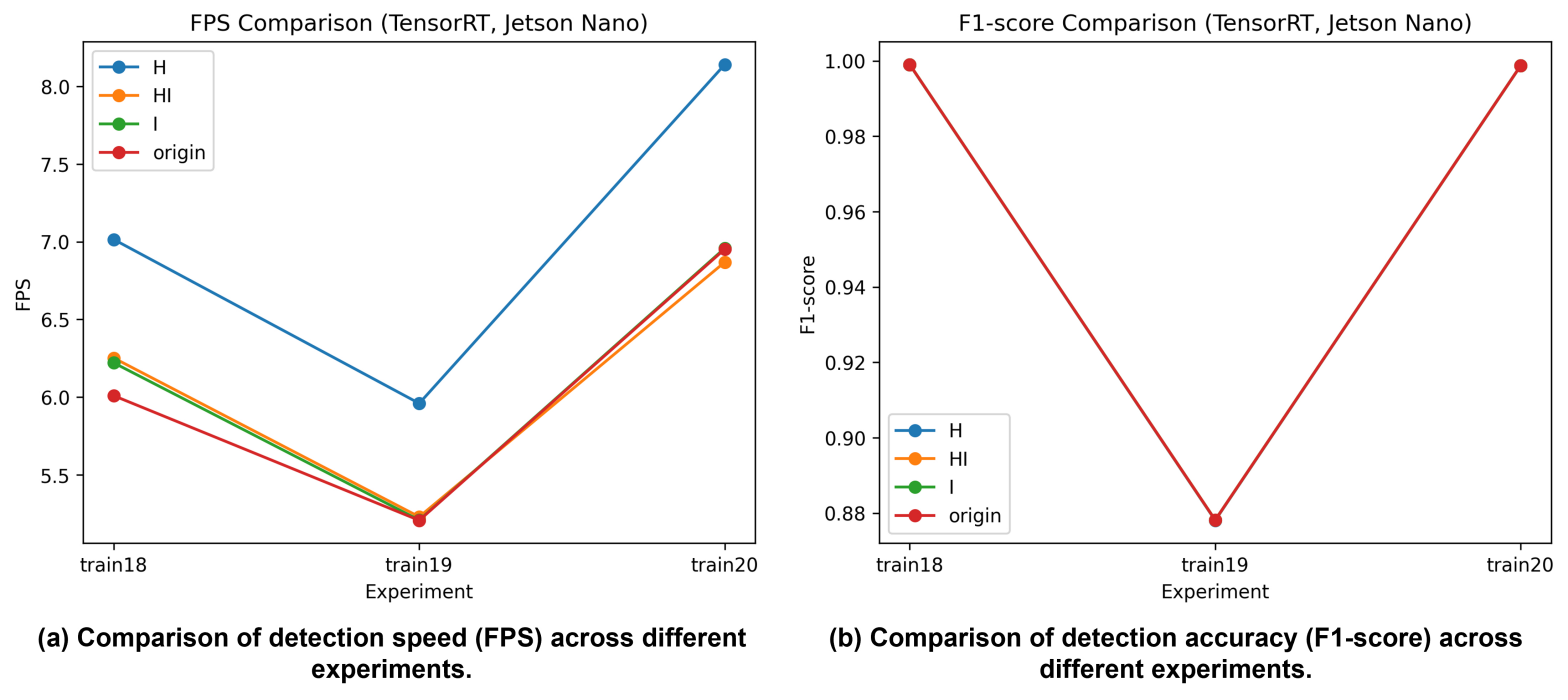
License plate detection performance using TensorRT on Jetson Nano: (**a**) detection speed (FPS); (**b**) detection accuracy (F1-score).

Detection performance on the Jetson Nano platform is evaluated using TensorRT-optimized models that exhibit high accuracy on the RTX 2080 Ti. As shown in Figure 8, precision, recall, and F1-score remain largely consistent across different TensorRT configurations (FP16, FP16 + INT8, INT8, and original), with variations limited to the third decimal place. This indicates that detection accuracy is dominated by dataset characteristics rather than hardware-level optimization.

Inference speed varies across configurations, with FP16 generally achieving the highest FPS on Jetson Nano. However, increased throughput does not lead to noticeable improvements in detection accuracy, confirming a speed–accuracy trade-off under embedded constraints. Notably, for the UFPR dataset, F1-scores remain relatively low regardless of FPS, further emphasizing the dominant impact of dataset difficulty on detection performance.

#### 4.3.5. Detection Performance on Raspberry Pi 5

**Figure 9:**
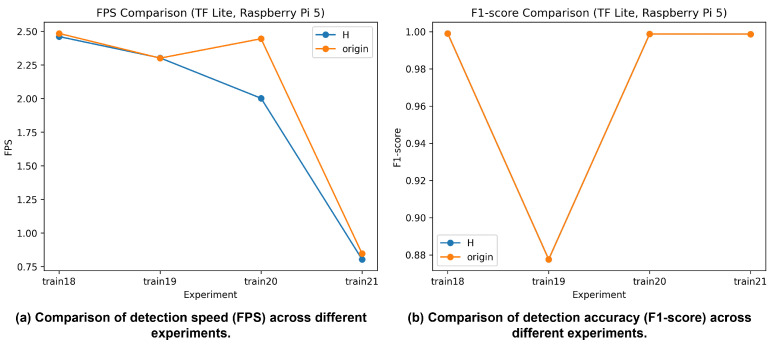
License plate detection performance using TensorFlow Lite on Raspberry Pi 5: (**a**) detection speed (FPS); (**b**) detection accuracy (F1-score).

Detection performance on the Raspberry Pi 5 platform is evaluated using TensorFlow Lite-based inference, as shown in Figure 9. Precision, recall, and F1-score remain nearly identical across the FP16 and original configurations, indicating that inference option changes have minimal impact on detection accuracy. In contrast, inference speed is slightly higher under the original configuration, suggesting a modest advantage in throughput.

Across datasets, the RodoSol-ALPR and AI-HUB datasets maintain highly stable detection performance with F1-scores exceeding 0.998, whereas the UFPR-ALPR dataset exhibits lower F1-scores in the range of approximately 0.88–0.92. This trend is consistent with results observed on the GPU and Jetson Nano platforms, reinforcing that dataset difficulty is the dominant factor influencing detection performance across hardware environments.

Overall, in low-power embedded settings such as the Raspberry Pi 5, inference configuration selection should prioritize processing speed over marginal accuracy differences. Based on the experimental results, the original configuration provides the most practical balance between detection accuracy and FPS for real-world deployment.

### 4.4. License Plate Recognition Results

This section presents a quantitative evaluation of license plate recognition (OCR) performance following the detection stage. Two OCR architectures are evaluated: (1) a CNN + Attention-LSTM-based model (Model 1, v4) and (2) a MobileNetV3 + Transformer decoder-based model (Model 2, v7). Each model is trained on three datasets (AI-HUB, RodoSol-ALPR, and UFPR-ALPR) and evaluated on a high-performance GPU (RTX 2080 Ti) as well as embedded platforms. Performance is measured using edit distance (ED), character accuracy (CA), character error rate (CER), and frames per second (FPS).

#### 4.4.1. License Plate Recognition Performance on RTX 2080 Ti

##### CNN + Attention-LSTM-Based OCR (Model 1, v4)

Table 4 summarizes the recognition performance of Model 1 on the RTX 2080 Ti. The model trained on the AI-HUB dataset (exp000) achieves the best overall performance, with an average ED of 0.0583, CA of 99.12%, and CER of 0.76%, while maintaining a high inference speed exceeding 200 FPS. These results demonstrate the effectiveness of large-scale, domain-specific data for Korean license plate recognition.

The model trained on the RodoSol-ALPR dataset (exp001) shows slightly reduced accuracy compared to AI-HUB, achieving a CA of 96.79% and CER of 3.21%, while maintaining an inference speed of approximately 170 FPS. This performance degradation is attributed to increased variability in imaging conditions and license plate appearance.

In contrast, the model trained on the UFPR-ALPR dataset (exp002) exhibits severely degraded recognition accuracy, with a CA of 24.18% and CER of 75.42%. Despite high FPS, the recognition performance is insufficient for practical use. This consistent degradation across runs indicates a dataset-imposed performance ceiling caused by extreme perspective distortion, small character sizes, and low effective resolution, rather than unstable optimization or stochastic training effects.

The large FPS gap observed between Model 1 and Model 2 on the RTX 2080 Ti is primarily attributed to differences in decoder behavior. Although Transformer decoders enable parallel attention computation, autoregressive decoding with a batch size of one still requires sequential token generation. In contrast, the simpler Attention-LSTM decoder incurs lower per-step overhead, resulting in substantially higher practical throughput.

##### MobileNetV3 + Transformer Decoder-Based OCR (Model 2, v7)

Table 5 summarizes the recognition performance of Model 2 on the RTX 2080 Ti. The model trained on the AI-HUB dataset (exp000) achieves competitive recognition accuracy, with a CA of 98.86% and a CER of 0.99%, indicating that the Transformer decoder effectively captures inter-character dependencies in Korean license plates. However, inference speed is substantially lower than that of Model 1, with FPS reduced to approximately 21 due to the higher computational and memory overhead of the Transformer-based decoder.

For the RodoSol-ALPR dataset (exp001), reducing the number of Transformer layers improves inference speed to approximately 26 FPS while preserving a CA of 96.30%, yielding the most balanced accuracy–speed trade-off among Model 2 configurations. In contrast, for the UFPR-ALPR dataset (exp002), increasing the number of Transformer layers fails to improve recognition accuracy and further reduces FPS to below 10. This behavior suggests that under severely degraded imaging and tracking conditions, Transformer-based sequence modeling provides limited benefits, and dataset difficulty dominates overall recognition performance.

#### 4.4.2. License Plate Recognition Performance on Jetson Nano

Jetson Nano represents a resource-constrained embedded environment in which processing speed becomes a critical factor for real-time OCR deployment. Table 6 summarizes the recognition performance of both OCR models on this platform.

Under this setting, Model 1 satisfies real-time requirements. The AI-HUB–trained model (exp000) achieves a CA of 99.10%, a CER of 0.77%, and an inference speed of approximately 22 FPS. The RodoSol-trained model (exp001) records the highest throughput, exceeding 23 FPS, while maintaining sufficient accuracy for alphanumeric license plate recognition. In contrast, although the UFPR-trained model (exp002) attains comparable FPS, its recognition accuracy remains extremely low, indicating that dataset difficulty dominates performance under severe imaging and tracking conditions.

Model 2 exhibits substantial performance degradation on Jetson Nano. As shown in Table 6b, inference speed drops to below 5 FPS across all datasets, with the UFPR-trained model falling below 2 FPS. These results indicate that, under the current autoregressive decoding setup, the Transformer-based v7 model does not meet real-time processing requirements on low-power embedded platforms. Consequently, its practical applicability in such environments is limited unless additional optimization strategies, such as decoder simplification, hardware-specific acceleration, or non-autoregressive inference, are employed.

#### 4.4.3. License Plate Recognition Performance on Raspberry Pi 5

Raspberry Pi 5 provides slightly higher computational capability than Jetson Nano but lacks dedicated GPU acceleration. Table 7 summarizes the recognition performance of both OCR models on this platform.

Model 1 continues to exhibit stable recognition performance. As shown in Table 7a, the AI-HUB–trained model (exp000) achieves a CA of 99.10% with an inference speed of approximately 16 FPS, which is sufficient for real-time OCR in low-power environments. Similar trends are observed on the RodoSol dataset, while recognition accuracy on the UFPR dataset remains consistently low, reaffirming that dataset difficulty dominates performance under severely degraded imaging conditions.

Model 2 again demonstrates clear throughput limitations on Raspberry Pi 5. According to Table 7b, the AI-HUB–trained model achieves approximately 5 FPS, while the RodoSol-trained model reaches around 9 FPS, approaching but not consistently meeting real-time requirements. Performance on the UFPR dataset remains inadequate in both accuracy and speed. These results confirm that Transformer-based decoders impose substantial computational overhead on embedded platforms without GPU acceleration, limiting their practical applicability under real-time constraints.

#### 4.4.4. Overall Analysis and Discussion

Although FPS values for detection and recognition modules are reported individually, they do not directly translate into end-to-end system throughput. In practical ALPR pipelines, overall latency is determined by the slowest module due to pipeline synchronization, memory transfers, and sequential execution constraints.

From a computational perspective, this observation is consistent with the model complexity analysis in Section 3.5. Despite having fewer parameters, the Transformer-based OCR model incurs higher FLOPs and memory usage, resulting in reduced practical efficiency. This discrepancy highlights that parameter count alone is an insufficient indicator of real-time suitability in deployment-oriented scenarios.

Results on the UFPR-ALPR dataset further clarify conditions under which OCR architecture choice becomes largely ineffective. When (1) the detected license plate region contains an insufficient number of effective pixels per character, (2) severe perspective distortion disrupts spatial alignment, and (3) tracking-based ROI propagation introduces cumulative spatial drift, both LSTM-based and Transformer-based decoders fail to recover reliable character sequences. Under such conditions, upstream feature degradation dominates overall performance, rendering decoder-level optimization insufficient.

Finally, the low variance observed across repeated runs indicates narrow confidence intervals for the reported mean recognition accuracies. As a result, the relative performance differences discussed in this section remain statistically meaningful, even without formal hypothesis testing.

### 4.5. Controlled Ablation Results with a Fixed ResNet-18 Backbone

Table 8 summarizes the OCR performance obtained under the controlled ablation setting, where the CNN backbone is fixed to ResNet-18 and only the sequence decoder is varied. For each dataset, the best-performing configuration among multiple training runs is reported.

All ablation configurations are trained three times using different random seeds. Across all datasets, the standard deviation of CA remains below ±0.5%, indicating that the observed performance trends are robust and not sensitive to stochastic training effects.

On the AI-HUB dataset, recognition accuracy is substantially lower than that of the primary OCR configurations, highlighting the importance of high-quality feature representations for Korean license plate recognition. In contrast, on the RodoSol-ALPR dataset, the ablation model achieves performance comparable to the main Attention-LSTM-based model, suggesting that decoder choice has a limited impact under relatively stable imaging conditions.

For the UFPR-ALPR dataset, recognition accuracy remains low despite the fixed backbone, confirming that performance is dominated by tracking-induced ROI instability and severe image degradation. These results demonstrate that in tracking-based scenarios, recognition failures are driven primarily by upstream errors in detection and ROI propagation, rather than by differences in sequence decoder architecture.

It should be noted that the term “tracking-induced ROI instability” used in this analysis does not refer to artifacts of a specific tracking algorithm. Rather, it denotes a general class of errors arising from sequential ROI propagation, including spatial jitter, gradual drift, and frozen region effects, which commonly occur in practical ALPR pipelines that rely on frame-to-frame ROI association.

#### Implications of the Controlled Ablation Study

The controlled ablation results provide critical context for interpreting the main comparative findings. When the CNN backbone is fixed, recognition performance still varies substantially across datasets, indicating that observed differences cannot be attributed to sequence decoder choice alone. Notably, severe performance degradation on tracking-based datasets persists regardless of decoder design, underscoring the dominant influence of ROI stability and detection quality. The substantial performance drop observed on the AI-HUB dataset under the controlled ablation setting should not be interpreted as evidence of bias or inadequacy in the sequence decoder itself. Rather, this degradation highlights the strong sensitivity of Korean license plate recognition to feature representation quality. By constraining the backbone to a fixed ResNet-18 configuration, the ablation intentionally limits feature capacity, thereby exposing an upper-bound proxy for decoder performance under suboptimal feature conditions. In this sense, the controlled ablation is designed to evaluate decoder robustness under shared and restricted representational constraints, rather than to reflect optimal end-to-end OCR performance. The results therefore indicate that decoder-level improvements alone cannot compensate for insufficient feature quality, particularly in complex, large-scale datasets such as AI-HUB.

These findings indicate that the benefits of advanced sequence modeling, such as Transformer-based decoding, are conditional on reliable feature extraction and stable ROI generation. As a result, improving OCR robustness in real-world traffic systems requires joint optimization of detection, tracking, and recognition stages rather than isolated refinement of the OCR decoder.

Importantly, the UFPR-ALPR dataset represents a class of realistic deployment scenarios characterized by mobile cameras, long-range capture, and tracking-based recognition. In such conditions, system-level degradation overwhelms decoder-level differences. Therefore, the limited differentiation between OCR architectures under UFPR conditions should be interpreted as evidence that recognition robustness must be addressed upstream, particularly at the detection and tracking stages.

### 4.6. Error Analysis of License Plate Recognition

Building on the failure patterns identified in the preceding analysis, this section presents a quantitative breakdown of error types to substantiate the observed qualitative trends and to clarify their dataset-dependent behavior. In this context, “tracking-induced” is used as a system-level descriptor of ROI propagation behavior over time, rather than as an attribution to any particular tracking implementation. Similar degradation patterns have been observed across different datasets and OCR architectures, suggesting that the identified failure modes are inherent to sequential ROI processing under unstable imaging conditions.

#### 4.6.1. Quantitative Analysis of Tracking-Induced Failures

The error analysis is conducted with explicit consideration of dataset-specific characteristics. Korean license plate–specific error categories, including prefix confusion and structural misalignment in mixed Korean–digit strings, are analyzed primarily using the AI-HUB dataset, which reflects real-world domestic traffic conditions.

Results from the RodoSol-ALPR dataset are treated separately as a cross-domain generalization case for Latin-based license plates under relatively stable imaging conditions. In contrast, the UFPR-ALPR dataset is used to analyze tracking-induced failure modes that arise from severe motion, perspective distortion, and ROI propagation errors, which are largely independent of language or plate format.

As shown in Table 9, the character error rate increases monotonically with increasing ROI instability. This relationship is consistently observed across both v4 and v7 models, indicating that tracking-induced ROI degradation constitutes a dominant performance bottleneck that cannot be resolved through decoder architecture alone.

Beyond general accuracy degradation, tracking-based datasets exhibit a distinct failure mode characterized by repeated identical OCR predictions across consecutive frames. This behavior reflects frozen or misaligned ROIs, in which the recognition model repeatedly processes an incorrect or nearly static input region, leading to persistent recognition errors.

Table 10 shows that repeated identical predictions occur with high frequency for both v4 and v7 models. This pattern indicates that a large portion of recognition failures originates from upstream ROI propagation errors rather than intrinsic limitations of the OCR architectures. The similar failure frequencies observed across models further support the conclusion that tracking noise dominates recognition performance in sequential datasets.

#### 4.6.2. Definition of Error Types

**Figure 10:**
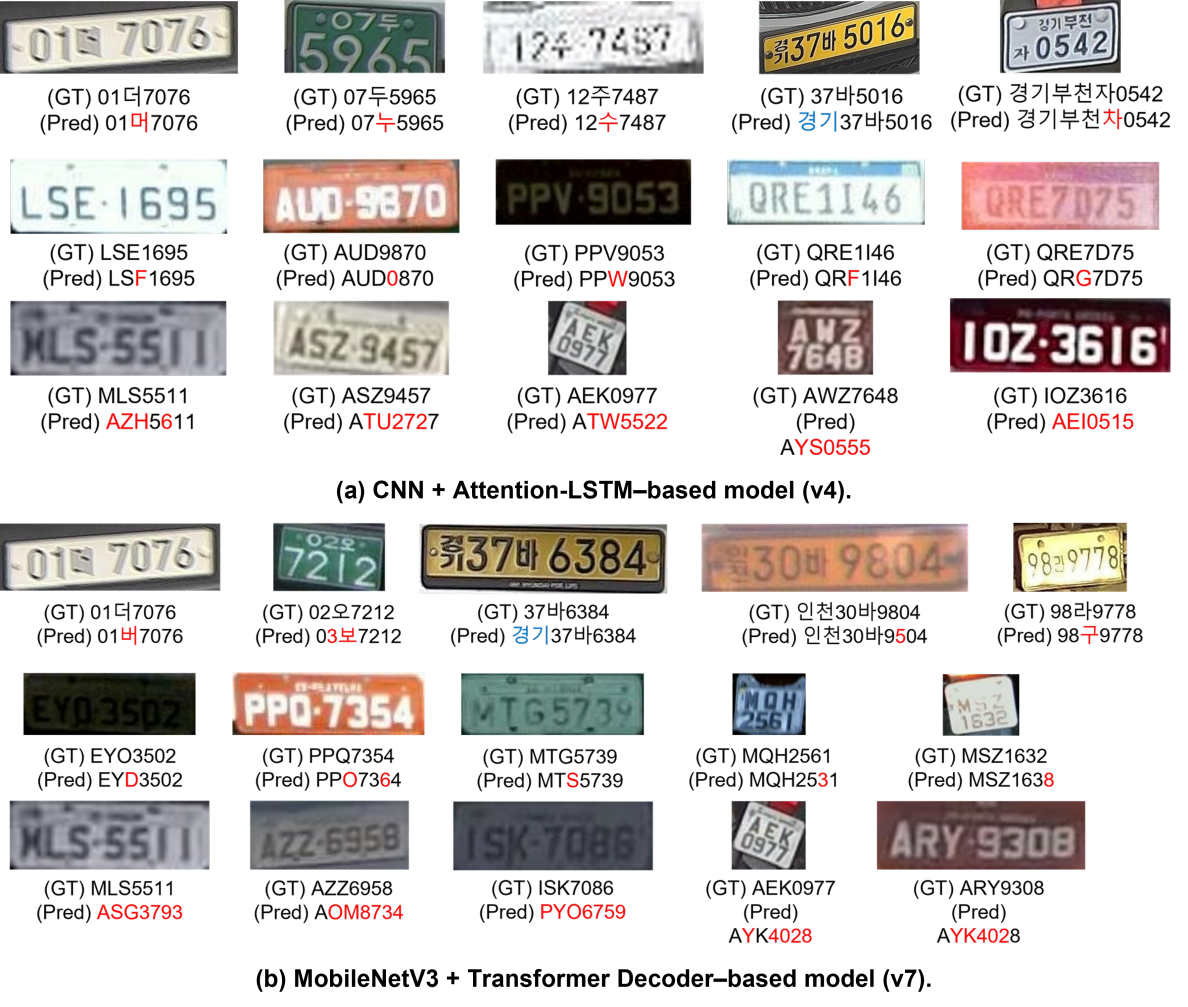
Representative misrecognition examples for the two OCR models. The upper row shows results from the CNN + Attention-LSTM-based model (v4), while the lower row corresponds to the MobileNetV3 + Transformer decoder-based model (v7). The examples illustrate dominant failure patterns, including repeated predictions and structural collapse, consistent with the quantitative analysis summarized in Table 10. Non-English characters correspond to Korean license plate characters from the original dataset and are preserved as part of the experimental data. Some regions are intentionally masked to protect personal information.

This subsection defines representative recognition error categories observed in the license plate recognition experiments. By analyzing typical failure cases, we examine how error patterns vary according to dataset characteristics and OCR model structures (v4 and v7). Error definitions are shared across all datasets (exp000, exp001, and exp002), while their frequencies and manifestations differ by dataset, as illustrated in Figure 10.

To enable systematic analysis, recognition errors are categorized into six types. Korean character confusion refers to misrecognition between visually similar Korean characters (e.g., “deo” vs. “meo”, “du” vs. “nu”, “ju” vs. “su”, “ja” vs. “cha”, “ra” vs. “gu”). These errors typically occur under low-resolution or blurred conditions, where fine-grained stroke information is lost during feature extraction.

Alphabet confusion denotes misrecognition among uppercase English letters with similar visual shapes, such as “E” vs. “F”, “Q” vs. “G”, “Z” vs. “S”, “H” vs. “S”, and “K” vs. “Y”. These errors are more common in license plates with low contrast or imbalanced character distributions.

Digit confusion arises from visually similar digits (e.g., “9” vs. “0”, “1” vs. “I”, “6” vs. “8”, “4” vs. “1”) and is often caused by font variations, strong reflections, or motion blur.

Prefix errors refer to omission, substitution, or over-recognition of regional prefixes (e.g., Gyeonggi, Incheon). For example, “37ba5016” may be misrecognized as “Gyeonggi37ba50”, or “GyeonggiBucheonja0542” as “GyeonggiBucheoncha0542”. Such errors are primarily attributed to variable string lengths and misalignment between detection and recognition stages.

Structural errors represent severe failures in which the overall string structure collapses (e.g., “MLS5511” recognized as “AZH5611”). These errors occur frequently in tracking-based datasets due to accumulated ROI misalignment or incorrect frame propagation.

Finally, annotation errors correspond to cases where the ground-truth labels are incorrect. These are treated as dataset quality issues rather than model limitations and are analyzed separately from recognition performance.

#### 4.6.3. Quantitative Breakdown of Error Types

Table 11 presents a quantitative summary of recognition error distributions across datasets and OCR models. The results confirm that the failure patterns illustrated in Figure 10 reflect consistent dataset-dependent trends rather than isolated examples.

On the AI-HUB dataset, recognition errors are dominated by Korean character confusions. This tendency is more pronounced for the v4 model, while the v7 model exhibits a reduced proportion of Korean-specific errors, indicating improved context modeling for mixed Korean–digit sequences. Other error types, including digit and structural errors, occur with relatively low frequency.

For the RodoSol-ALPR dataset, errors are primarily attributed to alphabet and digit confusions, which is consistent with its Latin-based alphanumeric license plate structure. Structural errors remain infrequent for both models, reflecting the stable imaging conditions and limited reliance on long-term tracking.

In contrast, the UFPR-ALPR dataset exhibits a fundamentally different error profile. For both v4 and v7 models, structural errors overwhelmingly dominate, while character-level confusions occur infrequently. This indicates that recognition failures on UFPR are driven mainly by tracking-induced ROI degradation and accumulated spatial misalignment, rather than by limitations of the OCR decoder architecture.

It should be emphasized that Korean character error categories are only applicable to datasets containing Korean characters, namely AI-HUB. Accordingly, results from the RodoSol-ALPR dataset are interpreted exclusively as a cross-domain generalization case for Latin-based license plates and are not used to infer Korean-specific recognition behavior.

#### 4.6.4. Error Characteristics of the CNN + Attention-LSTM-Based Model (v4)

The CNN + Attention-LSTM-based OCR model (v4) exhibits distinct error characteristics depending on dataset properties. On the AI-HUB dataset (exp000), Korean character confusion is the dominant failure mode. Errors frequently occur between characters with similar stroke-level structures, resulting in partial misrecognition while preserving the overall string layout. This behavior suggests that v4 maintains stable global sequence modeling but remains sensitive to fine-grained visual ambiguities in Korean characters.

On the RodoSol-ALPR dataset (exp001), error patterns shift toward alphabet and digit confusions. Misrecognitions typically involve visually similar Latin characters or digits, reflecting limited generalization when transitioning from Korean-centered training distributions to purely alphanumeric license plate formats.

In contrast, the UFPR-ALPR dataset (exp002) is dominated by structural errors. Because UFPR consists of tracking-based sequential frames, accumulated ROI misalignment and temporal drift significantly degrade recognition quality. Under these conditions, the v4 model fails to preserve the overall string structure, indicating high sensitivity to tracking-induced noise despite its stability in single-frame recognition scenarios.

#### 4.6.5. Error Characteristics of the MobileNetV3 + Transformer Decoder-Based Model (v7)

The MobileNetV3 + Transformer decoder-based OCR model (v7) generally exhibits improved global structural consistency compared to the Attention-LSTM-based v4 model, although dataset-dependent error patterns remain evident. On the AI-HUB dataset (exp000), the frequency of Korean character confusion is reduced relative to v4, indicating that Transformer-based sequence modeling more effectively captures long-range contextual dependencies. In particular, prefix-related errors are substantially mitigated, suggesting stronger preservation of overall license plate structure.

On the RodoSol-ALPR dataset (exp001), the v7 model largely maintains correct string-level structure, with most errors confined to fine-grained alphabet or digit confusions. These results indicate that while global context modeling is effective, discriminating between visually similar characters remains challenging under purely visual ambiguity.

In contrast, on the UFPR-ALPR dataset (exp002), severe structural failures persist and closely resemble those observed in v4. Repeated identical predictions across consecutive frames are also frequently observed. As these failure modes appear with comparable frequency in both architectures, they are attributed primarily to tracking-induced ROI instability rather than limitations of the Transformer-based decoder itself.

#### 4.6.6. Comparative Discussion: v4 vs. v7

A direct comparison between the two OCR architectures highlights clear but conditional performance differences. The v7 model demonstrates improved recognition of Korean characters and more reliable handling of regional prefixes compared to v4. These gains are attributed to the Transformer decoder’s ability to model longer-range dependencies and preserve global string structure, thereby reducing the likelihood that local misrecognitions escalate into full structural collapse.

In contrast, digit recognition stability shows no substantial difference between the two models. Moreover, both v4 and v7 exhibit severe vulnerability to structural errors on tracking-based datasets such as exp002, where accumulated ROI instability dominates recognition performance. Under these conditions, architectural differences in sequence modeling provide limited benefit.

Therefore, improvements observed on the AI-HUB dataset should be interpreted as evidence of enhanced modeling of Korean-specific linguistic and structural characteristics, rather than a universal advantage of Transformer-based decoding. Conversely, results on the RodoSol-ALPR dataset primarily reflect the generalization capability of OCR architectures to Latin-based license plate formats under relatively stable imaging conditions.

#### 4.6.7. Summary

In summary, the v7 model provides measurable improvements over v4 in Korean character recognition and regional prefix handling. These gains confirm the effectiveness of Transformer-based decoding in modeling Korean-specific structural dependencies. However, both models remain highly susceptible to structural collapse in tracking-based datasets, where recognition failures are dominated by accumulated errors from detection and ROI propagation rather than decoder design.

The quantitative error analysis further shows that Transformer-based decoding primarily reduces Korean character and prefix-related errors, while having limited impact on structural failures induced by unstable tracking. These findings indicate that overall license plate recognition performance is governed by system-level robustness rather than OCR architecture alone. Consequently, future research should prioritize integrated optimization across detection, tracking, and recognition stages instead of isolated improvements to OCR models.

### 4.7. Mitigation Experiment for Tracking-Induced Errors

To examine whether simple temporal strategies can mitigate tracking-induced OCR failures, a lightweight mitigation experiment based on temporal majority voting is conducted. For each tracked license plate sequence, OCR predictions within a fixed temporal window of N=5 consecutive frames are aggregated, and the most frequently predicted character at each position is selected as the final output. The window size was chosen empirically as the smallest value that consistently yielded stable accuracy improvements across tracking-based datasets, while avoiding excessive temporal smoothing or additional latency. This approach introduces negligible computational overhead and does not require any modification to the OCR architecture. Accordingly, the observed tracking-induced failures should be interpreted as consequences of sequential ROI propagation under noisy localization conditions, rather than as deficiencies of a particular tracking algorithm. This interpretation is consistent with the comparable trends observed across different OCR architectures and mitigation strategies. From a temporal perspective, majority voting over a window of *N* frames introduces an inherent delay of (N−1)/2 frames, which corresponds to two frames for the configuration used in this study (N=5). For this reason, temporal voting is evaluated as an optional post-processing step rather than as a mandatory component of a real-time ALPR pipeline. All mitigation experiments in this section are conducted under an offline evaluation setting, with the goal of analyzing relative robustness trends of different OCR architectures under tracking-based conditions. End-to-end latency optimization and strict real-time constraints are therefore outside the scope of this analysis and are treated as system-level design considerations.

Table 12 summarizes the effect of temporal majority voting on tracking-based OCR performance using the UFPR-ALPR dataset. For both v4 and v7 models, the application of temporal voting consistently improves character accuracy (CA), reduces character error rate (CER), and alleviates severe structural errors caused by tracking instability. Importantly, the relative improvement provided by temporal voting is comparable for both architectures, indicating that tracking-induced failures are largely independent of the sequence decoder design. These results further support the conclusion that structural recognition errors in sequential datasets are primarily driven by upstream ROI instability, and can be partially mitigated through simple temporal aggregation without altering the OCR model itself. Crucially, the application of temporal majority voting does not confer a Transformer-specific advantage. Both the CNN + Attention-LSTM model (v4) and the MobileNetV3 + Transformer model (v7) exhibit similar relative performance gains under temporal aggregation, and the fundamental performance gap between architectures remains unchanged. This confirms that the main conclusion of this study holds: under tracking-based sequential recognition, OCR performance is governed primarily by ROI stability and upstream system behavior rather than by the choice of sequence decoder.

### 4.8. Detailed Analysis of Transformer Decoder Parameters (Model 2)

This subsection analyzes how key structural parameters of the Transformer decoder—namely the number of decoder layers (num_layers) and attention heads (nhead)—affect recognition accuracy and processing speed in the MobileNetV3-based OCR model (v7). Although Transformer-based architectures provide strong sequence modeling capability through self-attention, their computational cost increases rapidly as decoder depth and width grow. Therefore, understanding the accuracy–efficiency trade-off induced by these parameters is essential for practical deployment.

In this analysis, only num_layers and nhead are systematically varied, while all other Transformer components, including feed-forward network dimensions, dropout rates, and decoding strategy, are kept fixed. This design choice is intentional, aiming to isolate the impact of decoder depth and attention parallelism on OCR performance and inference speed. Expanding the parameter sweep to additional hyperparameters would substantially enlarge the search space and obscure causal attribution of performance changes.

Given the short and bounded sequence length of license plate strings, decoder depth and multi-head attention were identified as the dominant architectural factors governing the accuracy–efficiency trade-off. Accordingly, this focused analysis provides a clear and interpretable assessment of how Transformer decoder capacity influences practical OCR performance under real-time constraints.

#### 4.8.1. Experimental Setup

All experiments were conducted using the AI-HUB Korean license plate dataset under identical training conditions and data splits. Only the Transformer decoder parameters were varied, while the CNN feature extractor was fixed to MobileNetV3-Large. The embedding dimension ($d_{\mathrm{model}}=28$) and maximum sequence length (max_len=16) were kept constant across all configurations.

The embedding dimension was selected based on a trade-off between representational capacity and computational efficiency. In preliminary experiments, larger embedding dimensions caused a disproportionate increase in memory footprint and decoder-side latency due to the quadratic complexity of self-attention, which was particularly problematic in embedded environments. Conversely, smaller dimensions ($d_{\mathrm{model}}\leq 16$) consistently exhibited underfitting behavior, including unstable convergence and degraded character accuracy. Accordingly, $d_{\mathrm{model}}=28$ was chosen as the smallest configuration that reliably avoids underfitting while remaining feasible for real-time embedded inference.

Evaluation metrics include edit distance (ED), character accuracy (CA), character error rate (CER), and processing speed measured in frames per second (FPS).

Six Transformer decoder configurations were evaluated. The number of decoder layers and attention heads were increased jointly (1/1, 2/2, 4/4, 7/7, and 14/14), along with a baseline configuration (6/7), to analyze the accuracy–efficiency trade-off as decoder capacity increases.

#### 4.8.2. Recognition Accuracy Analysis

Table 13 summarizes the recognition performance obtained with different Transformer decoder configurations. Overall, recognition accuracy is strongly influenced by decoder depth and width, but the relationship is non-monotonic.

The shallowest configuration (1/1) achieves an average edit distance (ED) of 0.0848 and a character accuracy (CA) of 98.74%, indicating limited representational capacity for modeling character dependencies. The 2/2 configuration does not improve performance and instead exhibits a slight degradation (ED of 0.0949 and CA of 98.61%), suggesting that shallow Transformer structures are insufficient to reliably capture contextual information in license plate sequences.

Clear performance gains emerge once the decoder depth increases to four layers. The 4/4 configuration reduces ED to 0.0794 and improves CA to 98.80%, indicating that moderate depth is necessary to effectively model both local and global dependencies. The best overall recognition accuracy is achieved with the 7/7 configuration, which attains an ED of 0.0775 and a CA of 98.84%.

Further increasing model complexity to the 14/14 configuration does not yield meaningful accuracy improvements. This saturation effect suggests that, beyond a certain depth, additional Transformer layers provide diminishing returns for license plate recognition, where sequence lengths are short and structural variability is limited.

#### 4.8.3. Processing Speed (FPS) Analysis

Processing speed exhibits a strong inverse relationship with Transformer decoder complexity. The shallow configurations (1/1 and 2/2) achieve the highest throughput, reaching approximately 67 and 49 FPS, respectively, which is sufficient for high-throughput real-time OCR applications.

As decoder depth and attention width increase, FPS decreases rapidly. The 4/4 configuration drops to approximately 32 FPS, while the 6/7 and 7/7 settings further reduce throughput to around 23 and 21 FPS. The most complex configuration (14/14) achieves only about 11 FPS, falling well below practical real-time requirements for license plate recognition.

This degradation is primarily caused by the quadratic computational complexity of self-attention and the increased memory traffic introduced by deeper decoder stacks. Under the autoregressive decoding setting with batch size one, these costs dominate runtime behavior, effectively limiting the benefits of architectural parallelism.

#### 4.8.4. Trade-Off Analysis and Optimal Configuration

Jointly considering recognition accuracy and processing speed, a clear accuracy–efficiency trade-off is observed. Shallow configurations (1/1 and 2/2) achieve high FPS but exhibit insufficient recognition accuracy for reliable license plate recognition. In contrast, very deep configurations (e.g., 14/14) provide only marginal accuracy improvements while incurring substantial computational overhead, resulting in severely reduced throughput.

Among the evaluated settings, the 7/7 configuration offers the most balanced compromise between accuracy and efficiency. It achieves approximately 98.8% CA with an average edit distance of 0.077 while maintaining an inference speed of around 20 FPS on GPU platforms. Accordingly, this configuration is adopted as the representative setting for the Transformer-based OCR model in subsequent experiments, as it provides stable recognition performance without excessive computational cost.

#### 4.8.5. Discussion

This analysis shows that increasing the structural complexity of Transformer-based OCR models does not necessarily lead to improved performance. For Korean license plate recognition, where character sequences are short and follow well-defined structural patterns, moderately deep Transformer decoders achieve the most favorable balance between accuracy and efficiency. Beyond this point, additional layers mainly increase computational cost without providing commensurate gains in recognition accuracy.

Importantly, the Transformer decoder in Model 2 was intentionally designed under strict computational and structural constraints rather than optimized for maximal representational capacity. The goal of this study is therefore not to demonstrate the theoretical superiority of Transformer architectures, but to assess their practical effectiveness and limitations in realistic, tracking-based license plate recognition systems, particularly under embedded deployment constraints.

## 5. Limitations

Despite the comprehensive evaluation conducted in this study, several limitations should be acknowledged.

First, structural collapse in tracking-based datasets is not fully resolved. In datasets such as UFPR-ALPR, license plate regions detected in individual frames are propagated through tracking. Small localization errors accumulated during this process were observed to severely degrade OCR performance, resulting in repeated identical predictions or complete structural breakdown of character sequences. These failures occur in both v4 and v7 models and are attributed primarily to error coupling across the detection–tracking–recognition pipeline rather than to intrinsic limitations of the OCR architectures. In addition, the specific quantitative impact of tracking-induced errors may vary depending on the choice of tracking algorithm and its parameter configuration. Different tracking strategies can influence the magnitude and temporal characteristics of ROI instability. However, the phenomena observed in this study—including ROI jitter, gradual spatial drift, and frozen region effects—are not unique to a particular tracking method. Rather, they represent general failure modes inherent to sequential ROI propagation in video-based ALPR pipelines. Accordingly, the main conclusions of this work should be interpreted as system-level observations. While alternative tracking implementations may shift absolute recognition accuracy, the dominance of ROI stability over decoder architecture in determining OCR performance under tracking-based conditions is expected to persist across practical ALPR system designs.

Second, improvements in digit recognition remain limited. Although the Transformer-based v7 model shows clear gains in Korean character and prefix recognition, confusions among visually similar digits (e.g., 6↔8, 9↔0) persist. These errors are mainly caused by license plate font characteristics, reflective materials, and illumination-induced distortions, which are not fully addressed by sequence modeling alone.

Third, ground-truth (GT) annotation quality affects the interpretation of some results. A small portion of the AI-HUB dataset contains mismatches between annotations and actual license plate content. While such cases were explicitly identified during qualitative analysis, residual annotation noise may still influence quantitative metrics and lead to conservative performance estimates.

Fourth, the scope of architectural exploration is limited. This study focuses on key Transformer decoder parameters, namely the number of layers (num_layers) and attention heads (nhead), while other factors such as embedding dimension, alternative positional encoding strategies, and interactions between decoder depth and CNN feature resolution are not exhaustively explored.

Finally, end-to-end latency is not quantitatively measured. Although detection and recognition modules are evaluated independently in terms of FPS, the overall system latency—including data transfer, tracking, and post-processing—is not reported, limiting direct conclusions about full pipeline throughput.

Future work will focus on mitigating tracking-induced error accumulation through confidence-triggered re-detection, ROI realignment, and lightweight temporal integration strategies.

## 6. Conclusions and Future Work

Consequently, the FPS values reported in this study should be interpreted as module-level performance indicators rather than absolute end-to-end system throughput. Because detection, tracking, and recognition stages are evaluated independently, the overall latency of a fully integrated ALPR pipeline may differ due to synchronization overhead, memory transfer, and sequential execution constraints. Addressing this limitation requires system-level benchmarking beyond isolated module evaluation.

Future work will therefore involve fully integrated pipeline benchmarking to quantitatively measure end-to-end latency and energy consumption under realistic traffic surveillance workloads.

### Future Work

Future research directions are summarized as follows:

First, end-to-end optimization jointly considering detection, tracking, and recognition stages is required. In particular, to mitigate error accumulation in tracking-based scenarios, future work will explore OCR models that incorporate temporal consistency constraints, confidence-triggered re-detection, or detection-aware recognition mechanisms.

Second, specialized techniques for digit and visually similar character recognition will be investigated. These include class-weight adjustment reflecting license plate–specific font characteristics, contrastive learning for commonly confused digit pairs (e.g., 6/8 and 9/0), and lightweight language-model-based post-processing to reduce character-level errors.

Third, improvements to evaluation frameworks that explicitly account for ground-truth (GT) quality are necessary. Automatic GT inconsistency detection and confidence-aware evaluation strategies may help disentangle model limitations from dataset annotation noise.

Finally, real-time system optimization for embedded environments will be a key focus. Lightweight model design and deployment optimization targeting low-power devices such as Jetson Nano and Raspberry Pi will be explored, with joint consideration of end-to-end FPS and energy efficiency to enhance practical applicability in real traffic infrastructure scenarios.

## Figures and Tables

**Table 1:** Comparison of the proposed OCR model architectures.

Aspect	CNN + Attention-LSTM	MobileNetV3 + Transformer
Feature extractor	ResNet-18	MobileNetV3
Sequence modeling	Attention + LSTM	Transformer decoder
Parallel processing	Limited	Decoder-level
Architectural complexity	Low	Higher

**Table 2:** Model size and computational complexity of OCR architectures.

Model	Params (M)	FLOPs (G)	Memory (MB)
CNN + Attention-LSTM (v4)	∼11.2	∼2.8	∼85
MobileNetV3 + Transformer (v7)	∼6.4	∼4.9	∼110
ResNet-18 + Attention-LSTM (Abl.)	∼11.2	∼2.8	∼85

**Table 3:** Training and inference configuration of the OCR system.

Setting	Description
Maximum sequence length	16 characters
OCR input resolution	96×320 RGB image
Loss function	Cross-entropy loss with label smoothing (ϵ=0.1)
Decoder type	Autoregressive sequence decoding
Decoding strategy	Greedy decoding during inference
Optimizer	Adam optimizer
Initial learning rate	$1\times 10^{-4}$
Training epochs	50 epochs
Batch size (training)	64
Data augmentation	Random brightness/contrast adjustment, Gaussian blur, random affine transformation
Inference batch size	1 (frame-wise processing)
Inference precision	FP32 (default), FP16 for GPU acceleration when supported
Deployment format	PyTorch for training, TensorRT/ONNX for optimized inference

**Table 4:** License plate recognition results of Model 1 (v4) on RTX 2080 Ti. Reported values represent the mean over three independent runs. Standard deviation was within ±0.3% for CA and ±0.4% for CER across all datasets. Arrows indicate whether higher (↑) or lower (↓) values are better. Bold values denote the best performance for each metric.

Experiment	Dataset	ED ↓	CA ↑	CER ↓	FPS ↑
exp000	AI-HUB	**0.0583**	**0.9912**	**0.0076**	**208.19**
exp001	RodoSol	0.2246	0.9679	0.0321	169.79
exp002	UFPR	**5.2794**	**0.2418**	**0.7542**	199.10

**Table 5:** License plate recognition results of Model 2 (v7) on RTX 2080 Ti. Reported values represent the mean over three independent runs. Arrows indicate whether higher (↑) or lower (↓) values are better. Bold values denote the best performance for each metric.

Experiment	Dataset	ED ↓	CA ↑	CER ↓	FPS ↑
exp000	AI-HUB	**0.0764**	**0.9886**	**0.0099**	20.94
exp001	RodoSol	0.2586	0.9630	0.0369	**25.95**
exp002	UFPR	**5.5617**	**0.1954**	**0.7945**	9.53

**Table 6:** License plate recognition results on Jetson Nano, where (**a**) corresponds to Model 1 (v4) and (**b**) corresponds to Model 2 (v7). Arrows indicate whether higher (↑) or lower (↓) values are better. Bold values denote the best performance for each metric.

(a) Model 1 (v4)					
Experiment	Dataset	Edit Distance ↓	CA ↑	CER ↓	FPS ↑
exp000	AI-HUB	**0.0596**	**0.9910**	**0.0077**	**22.16**
exp001	RodoSol	0.2395	0.9658	0.0342	**23.12**
exp002	UFPR	**5.2933**	**0.2406**	**0.7562**	**22.89**
**(b) Model 2 (v7) **					
Experiment	Dataset	Edit Distance ↓	CA ↑	CER ↓	FPS ↑
exp000	AI-HUB	0.0765	0.9882	0.0099	**1.48**
exp001	RodoSol	0.2632	0.9624	0.0376	**4.15**
exp002	UFPR	**5.5606**	**0.1962**	**0.7944**	**1.53**

**Table 7:** License plate recognition results on Raspberry Pi 5, where (**a**) corresponds to Model 1 (v4) and (**b**) corresponds to Model 2 (v7). Arrows indicate whether higher (↑) or lower (↓) values are better. Bold values denote the best performance for each metric.

(a) Model 1 (v4)					
Experiment	Dataset	Edit Distance ↓	CA ↑	CER ↓	FPS ↑
exp000	AI-HUB	**0.0596**	**0.9910**	**0.0077**	16.34
exp001	RodoSol	0.2395	0.9658	0.0342	16.81
exp002	UFPR	5.2933	0.2406	0.7562	16.65
**(b) Model 2 (v7)**					
Experiment	Dataset	Edit Distance ↓	CA ↑	CER ↓	FPS ↑
exp000	AI-HUB	0.0765	0.9882	0.0099	5.05
exp001	RodoSol	0.2632	0.9624	0.0376	9.26
exp002	UFPR	5.5606	0.1962	0.7944	4.90

**Table 8:** OCR performance under controlled ablation with a fixed ResNet-18 backbone. Arrows indicate whether higher (↑) or lower (↓) values are better.

Dataset	CA ↑	CER ↓	ED ↓	FPS ↑
AI-HUB (exp000_abl)	0.8939	0.1036	0.8496	140.94
RodoSol (exp001_abl)	0.9668	0.0332	0.2325	18.52
UFPR (exp002_abl)	0.4442	0.5509	3.8561	9.91

**Table 9:** Relationship between ROI quality and OCR error rate (CER) on tracking-based datasets.

ROI Quality Level	Jitter (px)	Drift (px)	CER
Stable ROI	<2	<5	Low
Moderate instability	2–5	5–15	Medium
Severe instability	>5	>15	High

**Table 10:** Frequency of tracking-specific failure modes on UFPR-ALPR dataset.

Failure Type	v4 (%)	v7 (%)
Repeated identical prediction	High	High
Frozen ROI over ≥3 frames	Moderate	Moderate
Structural collapse	High	High

**Table 11:** Distribution of error types across datasets and OCR models (%).

Dataset	Model	Korean	Alphabet	Digit	Structural
AI-HUB	v4	High	Low	Low	Very Low
AI-HUB	v7	Medium	Low	Low	Very Low
RodoSol	v4	Low	Medium	Medium	Low
RodoSol	v7	Low	Low	Medium	Low
UFPR	v4	Low	Low	Low	High
UFPR	v7	Low	Low	Low	High

**Table 12:** Effect of temporal majority voting on tracking-based OCR performance (UFPR-ALPR).

Model	CA	CER	Structural Errors
v4 (baseline)	Low	High	High
v4 + voting	Improved	Reduced	Reduced
v7 (baseline)	Low	High	High
v7 + voting	Improved	Reduced	Reduced

**Table 13:** License plate recognition performance according to Transformer decoder parameters (num_layers, nhead). Arrows indicate whether higher (↑) or lower (↓) values are better. Bold values denote the best performance for each metric.

Num_Layers/Nhead	ED ↓	CA ↑	CER ↓	FPS ↑
1/1	0.0848	0.9874	0.0110	67.42
2/2	0.0949	0.9861	0.0123	48.89
4/4	0.0794	0.9880	0.0103	32.26
6/7	0.0825	0.9879	0.0107	23.53
7/7	**0.0775**	**0.9884**	**0.0100**	20.86
14/14	0.0776	0.9883	0.0101	11.39

## Data Availability

The data that support the findings of this study are not publicly available due to privacy and legal restrictions but are available from the corresponding author upon reasonable request.
